# Supplementary material for: Serum 25(OH)D levels and mortality risk among middle-aged and elderly populations in the U.S.: A prospective cohort study
Source: PLoS One. 2025 Jul 24;20(7):e0328907. doi: 10.1371/journal.pone.0328907 (PMC12289007; doi:10.1371/journal.pone.0328907)
Supplement: S1 Table — (DOCX) [file pone.0328907.s002.docx]

**Serum vitamin D levels over the past 30 days among participants grouped by fish consumption levels in the NHANES 2001-2002 survey cycle**

| **^*^Eaten fish in the past 30 days** | **No** | **Yes** | **Standardize diff.** | **P-value** |
| --- | --- | --- | --- | --- |
| Number of participants | 86 | 202 |  |  |
| Vitamin D level (nmol/L) | 44.40 (30.20-67.60) | 57.50 (37.30-72.40) | 0.25 (-0.01, 0.50) | 0.021 |
|  |  |  |  |  |

Results in table: Median (Q1-Q3)

^*^The groups are divided by whether the individual has eaten any of the three fish (tuna, salmon, or sardines) in the past 30 days.
