# Supplementary material for: Serum 25(OH)D levels and mortality risk among middle-aged and elderly populations in the U.S.: A prospective cohort study
Source: PLoS One. 2025 Jul 24;20(7):e0328907. doi: 10.1371/journal.pone.0328907 (PMC12289007; doi:10.1371/journal.pone.0328907)
Supplement: Attachment — Submitted filename: Response to Reviewers.docx [file pone.0328907.s004.docx]

Responses to Reviewers

Manuscript Number: PONE-D-25-08106

Title: Serum 25(OH)D Levels and Mortality Risk Among Middle-Aged and Elderly Populations in the U.S.: A Prospective Cohort Study

Dear Editor/Reviewers:

Thank you very much for your valuable suggestions,which improved our manuscript.We responded to each comment and made corresponding changes in the manuscript.We believe that the revised manuscript is now much easier for readers to follow.According to these comments and suggestions,we added new information to the revised manuscript to help clarify the content.The response to each comment and corresponding changes are listed as follows.The comments from reviewers are shown in **blue**,and the responses are shown in **black**.The revisions in the revised manuscript shown in **red**. In addition.

Your efforts in reviewing the manuscript are highly appreciated.

Sincerely

Authors of PONE-D-25-08106

**Reviewer #1:**

**Comments:**

1- A well written comprehensive manuscript.
2- Relevant Comprehensive statistical test used.
3- Updated references used in the introduction and Discussion both
4- Results according to the study objectives
5- Conclusion aligned with the results

**Answer:** Thank you for reviewing our manuscript amidst your busy schedule and for your positive feedback on it!

**Reviewer #2:**

1. **Comments:** Numerous cohort studies and meta-analyses have already investigated the association between serum 25(OH)D levels and mortality risk in various populations. While the existing literature provides substantial evidence on this topic, it is essential to clarify the necessity and innovation of this study. Specifically, the authors should explicitly state how this study adds to the current body of knowledge.

**Answer:** Thank you for your suggestion. We supplement the necessity and innovation of the lack of research in this paper, which is mainly reflected in the following aspects:

Necessity: At present, there is no consensus on the diagnostic threshold of vitamin D deficiency and insufficiency. The mechanism of vitamin D in the prevention of all-cause death, cardiovascular disease and cancer is not clear, and further research is needed. The lack of vitamin D level standards for specific age groups (40-70 years old) limits the formulation of health management strategies.

Innovation: The nonlinear relationship between vitamin D level and mortality was proposed, and the inflection point was determined (for example, the inflection point of all-cause death was 54.80 nmol/L). The definition of existing vitamin D levels may be too strict, especially in some specific populations. It provides a more accurate diagnostic threshold of vitamin D for a specific age group, which helps to develop a more effective health management plan.

We have revised the content in the revised manuscript. (Revised manuscript: the Page 12, Line 247-256)

**Revised content:** This study indicates that there is a non-linear relationship between serum 25 (OH) D levels and all-cause mortality and idiopathic mortality in the middle-aged and elderly population aged 40-70 years. The inflection points corresponding to all-cause, CVD, and cancer mortality are 54.80 nmol/L, 44.70 nmol/L, and 58.70 nmol/L, respectively. However, there is currently no consensus on the optimal threshold for diagnosing vitamin D deficiency and insufficiency. For example, the American Institute of Medicine defines vitamin D deficiency as serum levels<30 nmol/L, while 30-50 nmol/L is considered vitamin D deficiency [13]. In contrast, the American Endocrine Society defines serum vitamin D levels below 50 nmol/L as deficiency and 50-75 nmol/L as insufficiency [14]. A meta-analysis of 62548 individuals in the general population showed that individuals with serum 25 (OH) D concentrations between 75-87.5 nmol/L had the lowest risk of mortality [56]. A cohort study from the UK Biobank showed that in the general population, the concentration of 25 (OH) D associated with the lowest risk of all-cause mortality was 60 nmol/L [57]. This indicates that the current threshold for defining serum 25 (OHD) adequacy may be too strict for certain populations. Therefore, our findings help determine the optimal threshold diagnosis for specific age groups, which is beneficial for developing health management plans.

**Reviewer #2:**

2. **Comments:** The abstract provides a summary of the key findings, including hazard ratios (HRs) for the association between serum 25(OH)D levels and mortality risk. However, to enhance the transparency and scientific rigor of the study, it is important to include the corresponding 95% confidence intervals (CIs) and p-values for the reported HRs.

**Answer:** Thank you for your careful review. Based on your comments, we have already added the corresponding 95% confidence interval (CI) and p-value for the HR report to the abstract.

We have revised the content in the revised manuscript. (Revised manuscript: the Page 2, Line 40-41)

**Revised content:** For all-cause mortality, the multivariate adjusted hazard ratios (HRs) across different 25(OH)D levels (< 25.00, 25.00 - 49.99, 50.00 - 74.99, and ≥ 75.00 nmol/L) were 1.00, 0.78 (0.65, 0.93) *p*=0.0069 , 0.59 (0.49,0.72) *p*<0.0001, and 0.54 (0.44, 0.66) *p*<0.0001 respectively.

**Reviewer #2:**

3. **Comments:** While the study presents hazard ratios (HRs) based on quartiles of serum 25(OH)D levels, it would be highly informative to quantify the linear association between continuous serum 25(OH)D levels and mortality risk. Specifically, the authors should consider analyzing and reporting the change in mortality risk per unit increase in serum 25(OH)D levels (e.g., per 10 nmol/L or ng/mL increase). This approach would provide a clearer understanding of the dose-response relationship and allow for more precise interpretation of the results. This analysis should be added to the results section, and its implications discussed in the context of existing evidence.

**Answer:** Thank you for your suggestion. Based on your suggestions, we identified through threshold effect analysis a nonlinear relationship between Serum 25(OH)D and mortality risk. The threshold points for all-cause mortality, CVD, and cancer were found to be 54.80 nmol/L, 44.70 nmol/L, and 58.70 nmol/L, respectively. Serum 25(OH)D levels showed a significant negative correlation with mortality risk below these threshold points, but no significant association was observed above these concentrations. We also created Table 3 to explain our results.

We have revised the content in the revised manuscript. (Revised manuscript: the Page 8, Line 175-183 and Page 8-9, Line 190-196)

**Revised content:** We further investigated the relationship between the risk of all-cause and cause-specific (CVD and cancer) mortality and 25(OH)D levels. As shown in Table 3, a nonlinear relationship was observed between 25(OH)D levels and mortality risk. When below 54.80 nmol/L, an increase of one unit in 25(OH)D concentration was associated with a 1.9% reduction in all-cause mortality (HR 0.981; 95% CI: 0.976, 0.985; p < 0.0001). When 25(OH)D levels exceeded 54.80 nmol/L, there was no significant association with all-cause mortality (HR 0.999; 95% CI: 0.996, 1.002; p = 0.6017). The inflection points for cause-specific mortality (CVD and cancer) were 44.70 nmol/L and 58.70 nmol/L, respectively. Below these inflection points, a one-unit increase in 25(OH)D concentration was associated with a 2.9% reduction in mortality for CVD (HR 0.971; 95% CI: 0.959, 0.983; p < 0.0001) and a 1.8% reduction for cancer (HR 0.982; 95% CI: 0.975, 0.990; p < 0.0001). When 25(OH)D levels exceeded these inflection points, there was no significant association with CVD mortality (HR 0.999; 95% CI: 0.994, 1.004; p = 0.7827) or cancer mortality (HR 1.004; 95% CI: 0.998, 1.009; p = 0.1798).

**Reviewer #2:**

4. **Comments:** The study has done a commendable job of adjusting for several important confounders in the multivariable models. However, two key variables—physical activity and caloric intake—are notably absent from Model 3. Physical activity is a well-known determinant of both vitamin D status (through outdoor activity and sunlight exposure) and mortality risk, while caloric intake reflects overall nutritional status and dietary patterns, which could also influence the observed association. To ensure a more comprehensive adjustment for potential confounding, it is recommended to include both physical activity and caloric intake as additional covariates in Model 3. This adjustment would further strengthen the validity and robustness of the study's conclusions.

**Answer:** Thank you for your suggestion. We have added covariates for physical activity and caloric intake. Physical activity was measured using the Global Physical Activity Questionnaire developed by the World Health Organization (WHO), while caloric intake was derived from participants' recall of food and beverage consumption in the 24 hours prior to the interview. Additionally, we have revised the baseline characteristics (Table 1), hazard ratios (95% CIs), subgroup analyses (Table 4), and survival curves along with smoothed curve-fitting model plots. The revised content also demonstrates the results.

We have revised the content in the revised manuscript. (Revised manuscript: the Page 4, Line 111-114, Page 4, Line 117-118, Page 5-6, Line 147-148, Page 7, Line 164-170)

**Revised content:** The assessment of physical activity was conducted using the Global Physical Activity Questionnaire (GPAQ) developed by the World Health Organization (WHO) [52]. The weekly amount of physical activity was calculated as metabolic equivalent minutes (MET minutes), with physical activity ≥ 600 MET minutes/week considered sufficient and physical activity<600 MET minutes/week considered insufficient [52].

Calorie intake data are derived from participants’ 24-hour food and beverage consumption recalled during interviews (from midnight to midnight).

**Fig 2. Dose–response associations of serum 25(OH)D level with risk of All-cause (a), CVD (b), Cancer (c) mortality.** The red solid line represents the estimated risk of all-cause and cause-specific mortality, with blue dashed lines showing 95 % CI, Analyses were adjusted for Gender, Race, Education, Martial Status, BMI Group, Drinking, Smoking, CVD, Caloric intake, Physical activity, Stroke, Hypertension, Diabetes, and PIR. PIR, ratio of family income to poverty; BMI, body mass index; CVD, cardiovascular disease

**Fig 3. Survival curves for all-cause and mortality by weighted Kaplan-Meier method with Log Rank test.**

**Fig 4. Survival curves for and Cardiovascular disease mortality by weighted Kaplan-Meier method with Log Rank test.**

**Reviewer #2:**

5. **Comments:** While the results section provides detailed statistical findings, the discussion and conclusion sections should more clearly and transparently summarize the key results to ensure they are accessible to readers. Specifically, the authors should explicitly restate the main findings, such as the magnitude of the association between serum 25(OH)D levels and mortality risk, and highlight any dose-response relationships or subgroup differences.

**Answer:** Thank you for your suggestion. In the first paragraph of the Discussion section, we re-described our findings. This study found that increasing serum 25(OH)D concentration significantly reduces all-cause, cardiovascular disease (CVD), and cancer mortality. The relationship between 25(OH)D concentration and mortality is non-linear, with protective effects observed only below specific thresholds for each cause of death.

We have revised the content in the revised manuscript. (Revised manuscript: the Page 11-12, Line 221-228)

**Revised content:** This study investigated the relationship between serum 25 (OH) D concentration and all-cause mortality, cardiovascular disease (CVD) mortality, and cancer mortality. Research has found that with an increase in serum 25 (OH) D concentration, all-cause mortality, CVD mortality, and cancer mortality all significantly decrease. Specifically, compared to the group with 25 (OH) D concentrations below 25.00 nmol/L, the group with concentrations ≥ 25.00 nmol/L showed a lower risk of mortality. Further analysis of the dose-response relationship revealed a non-linear relationship between 25 (OH) D concentration and mortality rate. When the concentration was below a specific threshold (such as 54.80 nmol/L, 44.70 nmol/L, and 58.70 nmol/L corresponding to all-cause, CVD, and cancer mortality rates, respectively), an increase in concentration significantly reduced the risk of mortality; And when the concentration exceeds these thresholds, this protective effect is no longer significant. The research results indicate that maintaining an appropriate concentration of 25 (OH) D may play an important role in reducing mortality.

**Reviewer #2:**

6. **Comments:** The discussion section should explicitly highlight the strengths of the study to provide a balanced perspective and underscore its contributions to the field.

**Answer:** Thank you for your suggestion. In the sixth paragraph of the discussion section, we highlighted the strengths of this study. Our research has a larger sample size and a longer follow-up period compared to similar studies. We also considered and adjusted for covariates such as participants' socioeconomic status, dietary habits, and lifestyle. Additionally, we used standardized methods to determine serum 25(OH)D concentrations in the NHANES database, ensuring the reliability of the data source.

We have revised the content in the revised manuscript. (Revised manuscript: the Page 13-14, Line 286-289)

**Revised content:** This study has three notable strengths. First, it has a large sample size and a long follow-up period, specifically examining the association between serum 25(OH)D concentration and mortality rates in the elderly population. Second, we carefully adjusted for socioeconomic status, dietary habits, lifestyle factors, comorbidities, and other potential confounders, further validating the reliability of our findings. Additionally, the use of standardized methods to determine serum 25(OH)D concentrations in the NHANES database ensures the reliability of our data sources.

**Reviewer #2:**

7. **Comments:** The study reports an unexpected finding that participants with severe vitamin D deficiency (the lowest quartile of serum 25(OH)D levels) had lower mortality rates compared to other quartiles. This observation contradicts the well-established literature, which generally associates lower vitamin D levels with higher mortality risk. The authors should address this finding in the discussion section and provide potential explanations.

**Answer:** Thank you for your suggestion. Our findings, like many relevant studies, support the association between lower vitamin D levels with higher mortality rates. In the section titled "Association of 25(OH)D concentration with all-cause and cause-specific mortality"(Page 7, Line 152-163), we constructed three Cox proportional hazards models. After adjusting for confounding factors, we found that the multivariable-adjusted hazard ratios (HRs) and 95% confidence intervals (CI) for the moderately deficient (25.0–49.9 nmol/L), insufficient (50.0–74.9 nmol/L), and sufficient (≥75.0 nmol/L) were all lower than those for the Severely deficient group (<25.0 nmol/L). This shows that compared with the severely deficient group, the other three groups have lower mortality. Additionally, we conducted a threshold effect analysis (Page 8-9, Line 172-184 and 191-198, ), revealing a nonlinear relationship between 25(OH)D concentration and mortality. When concentrations were below specific thresholds (e.g., 54.80 nmol/L for all-cause mortality, 44.70 nmol/L for cardiovascular disease mortality, and 58.70 nmol/L for cancer mortality), increasing concentrations significantly reduced mortality risks. However, when concentrations exceeded these thresholds, the protective effect was no longer significant. This observation is also well-reflected in the survival curves.

**Revised content:**

To explore the association between serum 25(OH)D levels and mortality, three Cox regression models were constructed. Multivariate adjustments included several factors: age, sex, race, education, marital status, body mass index, alcohol consumption, smoking, cardiovascular disease, stroke, hypertension, diabetes mellitus, and family income-poverty ratio. For all-cause mortality, the multivariate-adjusted hazard ratios (HRs) and 95% confidence intervals (CIs) across increasing levels of serum 25(OH)D (<25.00, 25.00–49.99, 50.00–74.99, and ≥75.00 nmol/L) were 1.00 (reference), 0.78 (0.65–0.93), 0.59 (0.49–0.72), and 0.54 (0.44–0.66) (Model 3). Similarly, for cardiovascular disease (CVD) mortality, the HRs and 95% CIs were 1.00 (reference), 0.71 (0.51–0.99), 0.55 (0.39–0.78), and 0.53 (0.36–0.77) across the four categories of serum 25(OH)D levels (Model 3). Additionally, for cancer mortality, the HRs and 95% CIs were 1.00 (reference), 0.85 (0.60–1.20), 0.62 (0.43-0.89), and 0.63 (0.42–0.92) across the same categories (Model 3). Compared with the group with serum 25(OH)D <25.00 nmol/L, middle-aged and older adults with higher levels of serum 25(OH)D (≥25.00 nmol/L) demonstrated significant associations with lower all-cause, CVD, and cancer mortality rates.

We further investigated the relationship between the risk of all-cause and cause-specific (CVD and cancer) mortality and 25(OH)D levels. As shown in Table 3, a nonlinear relationship was observed between 25(OH)D levels and mortality risk. When below 54.80 nmol/L, an increase of one unit in 25(OH)D concentration was associated with a 1.9% reduction in all-cause mortality (HR 0.981; 95% CI: 0.976, 0.985; p < 0.0001). When 25(OH)D levels exceeded 54.80 nmol/L, there was no significant association with all-cause mortality (HR 0.999; 95% CI: 0.996, 1.002; p = 0.6017). The inflection points for cause-specific mortality (CVD and cancer) were 44.70 nmol/L and 58.70 nmol/L, respectively. Below these inflection points, a one-unit increase in 25(OH)D concentration was associated with a 2.9% reduction in mortality for CVD (HR 0.971; 95% CI: 0.959, 0.983; p < 0.0001) and a 1.8% reduction for cancer (HR 0.982; 95% CI: 0.975, 0.990; p < 0.0001). When 25(OH)D levels exceeded these inflection points, there was no significant association with CVD mortality (HR 0.999; 95% CI: 0.994, 1.004; p = 0.7827) or cancer mortality (HR 1.004; 95% CI: 0.998, 1.009; p = 0.1798).

**Fig 3. Survival curves for all-cause and mortality by weighted Kaplan-Meier method with Log Rank test.**

**Fig 4. Survival curves for and Cardiovascular disease mortality by weighted Kaplan-Meier method with Log Rank test.**

We tried our best to improve the manuscript and we use blue to indicate the newly added content and red and the strikethrough to indicate the deleted content. which will not influence the content and framework of the paper.We appreciate for Editor/Reviewers' warm work earnestly,and hope the correction will meet with approval. Once again,thank you very much for your comments and suggestions.

Yours sincerely

Xiaopeng He

2025/4/26

Zhejiang Provincial People’s Hospital
