# Supplementary material for: Serum 25(OH)D levels and mortality risk among middle-aged and elderly populations in the U.S.: A prospective cohort study
Source: PLoS One. 2025 Jul 24;20(7):e0328907. doi: 10.1371/journal.pone.0328907 (PMC12289007; doi:10.1371/journal.pone.0328907)
Supplement: Attachment — Submitted filename: Responses to Reviewers.docx [file pone.0328907.s005.docx]

Responses to Reviewers

Manuscript Number: PONE-D-25-08106R1

Title: Serum 25(OH)D Levels and Mortality Risk Among Middle-Aged and Elderly Populations in the U.S.: A Prospective Cohort Study

Dear Editor/Reviewers:

Thank you for your thoughtful review and constructive feedback on our manuscript (Manuscript ID: PONE-D-25-08106R1). We appreciate the opportunity to revise and improve our work. Below, we have addressed each of your comments point by point. The comments from reviewers are shown in **blue**,and the responses are shown in **black**.The revisions in the revised manuscript shown in **green**. In addition. Your efforts in reviewing the manuscript are highly appreciated!

Sincerely

Authors of PONE-D-25-08106R1

**Reviewer #1:**

**Comments:** The standard deviation of calorie intake is notably high. Would it be more appropriate to present this data as the median with interquartile range (IQR) using the Kruskal-Wallis test instead?

**Answer:** We tested the normality of participants' calorie intake and found that it did not follow a normal distribution. Therefore, we presented the data as median (Q1, Q3).

**Table 1. Baseline characteristics of middle-aged and older adult by serum 25(OH)D concentration in NHANES 2001-2018**

|  | **Severely deficient** | **Moderately deficient** | **Insufficient** | **Sufficient** | ***p* value** |
| --- | --- | --- | --- | --- | --- |
| **Caloric intake^c^ (kcal)** | 1779.00 (1314.00-2346.00) | 1874.00 (1376.00-2524.00) | 1957.00 (1449.50-2607.00) | 1927.50 (1438.00-2552.00) | <0.001 |

^c^The continuous variables were found to be non-normally distributed and were therefore presented as median (quartile 1, quartile 3).

**Comments:** If available, it would be helpful to elaborate on the consumption of salmon, milk, and its products (which are rich in vitamin D) to better understand how participants could have such low serum vitamin D levels.

**Answer:** For foods rich in vitamin D, in the NHANES database, we only found data on seafood consumption from the 2001-2002 survey cycle, which included whether participants had consumed seafood in the past 30 days and the frequency of consumption. We selected three types of fish rich in vitamin D—tuna, salmon, and sardines—and compared the serum vitamin D levels between participants who had consumed any of these fish in the past 30 days and those who had not. Among the 10,477 participants in the 2001-2002 survey cycle, a total of 2,155 were matched to our study population. After excluding 1,867 individuals with missing data, 288 participants were included in the statistical analysis. The dataset included a total of 288 participants, with 86 reporting no fish consumption in the past 30 days and 202 having consumed at least one type of fish. We found that the serum vitamin D levels in the fish-consuming group were significantly higher than those in the non-fish-consuming group (p=0.021) (Support information S2 File).

**S2 File. Fish consumption compared to serum vitamin D levels over the past 30 days**

| **^*^Eaten fish in the past 30 days** | **No** | **Yes** | **Standardize diff.** | ***p* value** |
| --- | --- | --- | --- | --- |
| Number of participants | 86 | 202 |  |  |
| Vitamin D level (nmol/L) | 44.40 (30.20-67.60) | 57.50 (37.30-72.40) | 0.25 (-0.01, 0.50) | 0.021 |
|  |  |  |  |  |

Results in table: Median (Q1-Q3)

^*^The groups are divided by whether the individual has eaten any of the three fish (tuna, salmon, or sardines) in the past 30 days.

**Comments:** Furthermore, could we discuss the possible mechanisms linking vitamin D deficiency to cancer mortality? Is there any possible connection related to the co-absorption of calcium?

**Answer:** Our study indicates that compared to the group with severe vitamin D deficiency, the groups with insufficient and sufficient serum vitamin D levels have lower cancer mortality rates. We speculate that the possible mechanisms underlying the correlation between vitamin D levels and cancer mortality are as follows: Firslyt, calcitriol interferes with the growth and aggressiveness of cancer cells by promoting the differentiation of epithelial cells; These cells act as barriers, interfering with the migration of cancer cells through the extracellular matrix [61]. Secondly, calcitriol can also inhibit the proliferation of cancer cells by inducing G1 cell cycle arrest. As the promoter region of the cell cycle inhibitor p21 contains vitamin D response elements, calcitriol can directly regulate the transcription of the p21 gene, thereby exerting anti-tumor cell proliferation activity [62]. Thirdly, active vitamin D inhibits Wnt/β-catenin signaling through multiple pathways [67]. Abnormal activation of the Wnt/β-catenin signaling pathway has been widely observed in various cancers. Its overactivity can promote tumor growth, metastasis and treatment tolerance by maintaining the self-renewal ability of cancer stem cells [67]. For instance, in colorectal cancer, mutations in the APC gene lead to uncontrolled Wnt signaling, causing excessive accumulation of β-catenin and subsequently activating oncogene expression [61]. Vitamin D inhibits the abnormal activation of the Wnt/β-catenin signaling pathway by promoting the binding of VDR to β-catenin and reducing the binding of β-catenin to TCF [61]. Vitamin D upregulates the expression of the CDH1 gene (encoding E-cadherin), promoting E-cadherin to capture β-catenin and preventing it from entering the nucleus to activate downstream oncogenes [68]. Vitamin D also exerts an anti-cancer effect in cancers such as colorectal cancer by increasing the secretion of the Wnt inhibitor DKK-1, further inhibiting the abnormal activation of the Wnt signaling pathway [69]. Fourth, calcitriol exhibits anti-angiogenic effects in both in vitro and in vivo models by inhibiting the growth of tumor-derived endothelial cells (TDECs), reducing endothelial cell germination and morphogenesis, and lowering the expression of vascular endothelial growth factor (VEGF) and the number of microvessels [63]. Moreover, this effect depends on the vitamin D receptor (VDR) pathway. For instance, the levels of angiogenic factors (such as HIF-1α, VEGF, etc.) increase in VDR knockout mice, while calcitriol can inhibit VEGF-dependent tumor growth in normal mice [64]. Although calcitriol may induce VEGF expression in some cases, its overall anti-tumor effect may stem from the balance of pro-angiogenic and anti-angiogenic factors (such as simultaneous regulation of VEGF and Thrombospondin-1), rather than simply inhibiting angiogenesis [65,66]. In addition, vitamin D can promote the absorption of calcium ions and increase the intracellular calcium ion concentration [60], and an increase in intracellular calcium concentration can trigger apoptosis in various types of cancer cells [58,59]. For instance, breast cancer cells undergo programmed cell death after a continuous inflow of calcium stimulated by 1,25 (OH) 2D3. In contrast, normal breast cells have a reduced susceptibility to calcium-induced apoptosis, which may be due to the enhanced cytoplasmic calcium buffering mechanism.

**Reference**

58. I.S. Mathiasen, I.N. Sergeev, L. Bastholm, F. Elling, A.W. Norman, M. Jaattela, Calcium and calpain as key mediators of apoptosis-like death induced by vitamin D compounds in breast cancer cells, J. Bi	ol. Chem. 277 (2002) 3078–30745.
59. D.L. McConkey, S. Orrenius, The role of calcium in regulation of apoptosis, Biochem. Biophys. Res. Commun. 239 (1997) 357–366.
60. Sergeev IN. Calcium as a mediator of 1,25-dihydroxyvitamin D3-induced apoptosis. The Journal of Steroid Biochemistry and Molecular Biology. 2004 May 1;89-90:419–25.
61. Na SY, Kim KB, Lim YJ, Song HJ. Vitamin D and Colorectal Cancer: Current Perspectives and Future Directions. Journal of Cancer Prevention. 2022 Sep 30;27(3):147–56.
62. Scaglione-Sewell BA, Bissonnette M, Skarosi S, Abraham C, Brasitus TA. A vitamin D3 analog induces a G1-phase arrest in CaCo-2 cells by inhibiting cdk2 and cdk6: roles of cyclin E, p21Waf1, and p27Kip1. Endocrinology. 2000;141:3931–9. doi: 10.1210/endo.141.11.7782
63. Bernardi R.J., Johnson C.S., Modzelewski R.A., Trump D.L. Antiproliferative effects of 1α,25-dihydroxyvitamin D3 and vitamin D analogs on tumor-derived endothelial cells. Endocrinology. 2002;143:2508–2514. doi: 10.1210/endo.143.7.8887
64. Chung I., Han G., Seshadri M., Gillard B.M., Yu W.D., Foster B.A., Trump D.L., Johnson C.S. Role of vitamin D receptor in the antiproliferative effects of calcitriol in tumor-derived endothelial cells and tumor angiogenesis in vivo. Cancer Res. 2009;69:967–975. doi: 10.1158/0008-5472.CAN-08-2307
65. Garcia-Quiroz J., Rivas-Suarez M., Garcia-Becerra R., Barrera D., Martinez-Reza I., Ordaz-Rosado D., Santos-Martinez N., Villanueva O., Santos-Cuevas C.L., Avila E., et al. Calcitriol reduces thrombospondin-1 and increases vascular endothelial growth factor in breast cancer cells: Implications for tumor angiogenesis. J. Steroid Biochem. Mol. Biol. 2014;144:215–222. doi: 10.1016/j.jsbmb.2013.09.019
66. Grundmann M., Haidar M., Placzko S., Niendorf R., Darashchonak N., Hubel C.A., von Versen-Hoynck F. Vitamin D improves the angiogenic properties of endothelial progenitor cells. Am. J. Physiol. Cell Physiol. 2012;303:C954–C962. doi: 10.1152/ajpcell.00030.2012
67. Byers SW, Rowlands T, Beildeck M, Bong YS. Mechanism of action of vitamin D and the vitamin D receptor in colorectal cancer prevention and treatment. Rev Endocr Metab Disord. 2012;13:31–8. doi: 10.1007/s11154-011-9196-y
68. Razak S, Afsar T, Almajwal A, Alam I, Jahan S. Growth inhibition and apoptosis in colorectal cancer cells induced by Vitamin D-Nanoemulsion (NVD): involvement of Wnt/β-catenin and other signal transduction pathways. Cell Biosci. 2019;9:15. doi: 10.1186/s13578-019-0277-z.a877dbbaf57041a1be6b62a0f5acc464
69. Beildeck ME, Islam M, Shah S, Welsh J, Byers SW. Control of TCF-4 expression by VDR and vitamin D in the mouse mammary gland and colorectal cancer cell lines. PLoS One. 2009;4:e7872. doi: 10.1371/journal.pone.0007872
